# The First Two Complete Mitochondrial Genomes for the Subfamily Meligethinae (Coleoptera: Nitidulidae) and Implications for the Higher Phylogeny of Nitidulidae

**DOI:** 10.3390/insects15010057

**Published:** 2024-01-12

**Authors:** Jiaqi Dai, Meike Liu, Andrea Di Giulio, Simone Sabatelli, Wenkai Wang, Paolo Audisio

**Affiliations:** 1 Institute of Entomology, College of Agriculture, Yangtze University, Jingzhou 434025, China; 2021710713@yangtzeu.edu.cn; 2 MARA Key Laboratory of Sustainable Crop Production in the Middle Reaches of the Yangtze River (Co-Construction by Ministry and Province), College of Agriculture, Yangtze University, Jingzhou 434025, China; w_wenkai@hotmail.com; 3 Department of Science, Roma Tre University, Viale Guglielmo Marconi, 00146 Rome, Italy; andrea.digiulio@uniroma3.it; 4 Department of Biology and Biotechnologies “Charles Darwin”, Sapienza University of Rome, Viale dell’Università 32, 00185 Rome, Italy; simone.sabatelli@uniroma1.it (S.S.); paolo.audisio@uniroma1.it (P.A.)

**Keywords:** pollen beetle, species-specific markers, identification, evolution rate, barcode gene

## Abstract

**Simple Summary:**

The phylogenetic status of the family Nitidulidae and its sister group relationship remain controversial. Also, the phylogenetic status of the subfamily Meligethinae and its phylogenetic relationships with other subfamilies of Nitidulidae are not fully understood. Mitochondrial genome sequences can be used to study species identification, phylogeny, and population genetic structure, and to provide valuable molecular markers for further genetic studies. In this paper, two complete mitochondrial genomes of Meligethinae were provided for the first time, and the phylogenetic status of the family Nitidulidae and subfamily Meligethinae were explored based on the complete mitochondrial genomes. A comparative analysis of the general characteristics and non-coding region patterns of the complete mitochondrial genomes of *Meligethinus tschungseni* and *Brassicogethes affinis* revealed that the base composition and mitochondrial genome structure of these two species are markedly different. Given the results of the phylogenetic analysis based on 20 mitochondrial genomes, the status of Nitidulidae and its sister group relationship is discussed. We also attempted to analyze the taxonomic status of Meligethinae and its sister group relationship. This study will provide a basis for further studies on the higher phylogeny of Nitidulidae.

**Abstract:**

The phylogenetic status of the family Nitidulidae and its sister group relationship remain controversial. Also, the status of the subfamily Meligethinae is not fully understood, and previous studies have been mainly based on morphology, molecular fragments, and biological habits, rather than the analysis of the complete mitochondrial genome. Up to now, there has been no complete mitochondrial genome report of Meligethinae. In this study, the complete mitochondrial genomes of *Meligethinus tschungseni* and *Brassicogethes affinis* (both from China) were provided, and they were compared with the existing complete mitochondrial genomes of Nitidulidae. The phylogenetic analysis among 20 species of Coleoptera was reconstructed via PhyloBayes analysis and Maximum likelihood (ML) analysis, respectively. The results showed that the full lengths of *Meligethinus tschungseni* and *Brassicogethes affinis* were 15,783 bp and 16,622 bp, and the AT contents were 77% and 76.7%, respectively. Each complete mitochondrial genome contains 13 protein-coding genes (PCGs), 22 transfer RNA genes (tRNAs), 2 ribosomal RNA genes (rRNAs), and a control region (A + T-rich region). All the PCGs begin with the standard start codon ATN (ATA, ATT, ATG, ATC). All the PCGs terminate with a complete terminal codon, TAA or TAG, except *cox1*, *cox2*, *nad4*, and *nad5*, which terminate with a single T. Furthermore, all the tRNAs have a typical clover-leaf secondary structure except *trnS1*, whose DHU arm is missing in both species. The two newly sequenced species have different numbers and lengths of tandem repeat regions in their control regions. Based on the genetic distance and Ka/Ks analysis, *nad6* showed a higher variability and faster evolutionary rate. Based on the available complete mitochondrial genomes, the results showed that the four subfamilies (Nitidulinae, Meligethinae, Carpophilinae, Epuraeinae) of Nitidulidae formed a monophyletic group and further supported the sister group relationship of Nitidulidae + Kateretidae. In addition, the taxonomic status of Meligethinae and the sister group relationship between Meligethinae and Nitidulinae (the latter as currently circumscribed) were also preliminarily explored.

## 1. Introduction

Nitidulidae includes 11 subfamilies with approximately 350 genera and nearly 4500 species worldwide [1,2,3]. Meligethinae (Coleoptera: Nitidulidae) is the second largest subfamily of Nitidulidae, also known as “pollen beetles”, with 46 genera and approximately 700 species worldwide and 12 genera and approximately 130 species in China [4,5,6,7,8,9,10,11,12,13,14,15]. Meligethinae is widely distributed in the Nearctic, Afrotropical, Oriental, and Palaearctic realms, and (very marginally) in Australia, except the Neotropical realm [4,16]. It is worth noting that Meligethinae is the only subfamily among Nitidulidae that independently and entirely became strictly anthophagous, with all members of this lineage using pollen as the main food resource [17,18,19,20,21,22]. Meligethinae represent an important group to reveal the different and regular interactions between morphological structure, biological habit, and ecological adaptability among the various subfamilies of the family Nitidulidae [1,4,5,7,11,12,14,15,16]. The first two complete mitochondrial genomes of the subfamily Meligethinae analyzed here are *Meligethinus tschungseni* Kirejtshuk, 1987 and *Brassicogethes affinis* Jelínek, 1982, collected from palm flowers and rape flowers in China, respectively [23,24].

There are many studies on the status of Nitidulidae: Bocak et al. (2014) [25] supported Passandridae nested within Nitidulidae, and Tang et al. (2019) [26] supported Nitidulidae nested within Erotylidae based on the mitochondrial genome. Other studies supported that Nitidulidae is monophyletic based on morphological and molecular data analysis [1,3,27,28,29,30]. However, previous analyses based on the mitochondrial genome had a small sample size, without a complete mitochondrial genome of Meligethinae. Regarding the sister group relationship of Nitidulidae, most studies supported the sister group relationship of Nitidulidae + Kateretidae based on morphological characters [7,31,32]. The following studies supported the sister group relationship of Nitidulidae + Kateretidae: Cline et al. (2014) [28], based on seven molecular fragments (*12S*, *16S*, *18S*, *28S*, *COI*, *COII*, and *H3*); Bocak et al. (2014) [25], based on four molecular fragments (*18S*, *28S*, *rrnL*, and *COI*); Robertson et al. (2015) [2], based on eight molecular fragments (*18S*, *28S*, *H3*, *CAD*, *12S*, *16S*, *COI*, and *COII*); and Cai et al. (2022) [3], based on single-copy nuclear protein-coding (NPC) genes and fossil data. Only the phylogenetic trees constructed by Chen et al. (2020) [29] based on the complete mitochondrial genomes of 17 species (seven species of Nitidulidae and ten species of other Coleoptera) supported that the sister group of Nitidulidae could be Monotomidae, but this clade had low bootstrap support values in ML trees. In fact, this potentially spurious sister group relationship of (Nitidulidae + Monotomidae) was probably due to the mismatch between the dataset and the selected nucleotide substitution model [33].

Meligethinae, as the second largest subfamily in Nitidulidae, has always attracted much attention. Many scholars have used morphological characteristics and a small amount of molecular data to explore the taxonomic status of Meligethinae. Kirejtshuk et al. (1982, 1986, 1995, 2008) [34,35,36,37] supported Meligethinae as monophyletic based on a few morphological characters of the adults and biological habits such as larval host plants. Trizzino et al. (2009) [38] and Audisio et al. (2009) [39] also supported that Meligethinae is monophyletic based on morphological characters, molecular fragments, and larval host plants, respectively. Cline et al. (2014) [28] reconstructed the phylogenetic relationships among Nitidulidae based on seven molecular fragments (*12S*, *16S*, *18S*, *28S*, *COI*, *COII*, *H3*) and showed that Meligethinae nested in Nitidulinae, but Cline et al. (2014) only selected one species as a representative of Meligethinae. Lee et al. (2020) [1] reconstructed the phylogenetic relationships of Nitidulidae based on five molecular fragments (*COI*, *28S*, *CAD*, *H3*, Wingless), and proposed that Meligethinae (represented by three genera and seven species) is monophyletic, but also showed that Meligethinae nested in Nitidulinae. The phylogenetic trees constructed by Cline et al. and Lee et al. based on molecular fragments were insufficient to resolve the status of Meligethinae and its phylogenetic relationship with Nitidulinae (which, as presently circumscribed, very likely represent a polyphyletic lineage). Therefore, there is an urgent need to supplement new representative genera and species as well as molecular data (such as complete mitochondrial genomes) to continue studying Meligethinae. 

In recent years, the complete mitochondrial genome has been widely used to study the phylogenetic relationships among insects [40,41,42], phylogeography [43], and molecular evolution [40,44]. The mitochondrial genome of insects has unique features, such as maternal inheritance, rapid evolution rate, stable gene composition, and high independence and integrity, making it a powerful genetic marker for studying the evolution of insects [45,46]. Previously, there were only seven complete mitochondrial genomes among three subfamilies of Nitidulidae in GenBank [29,47,48,49]. Meligethinae, as the second largest subfamily of Nitidulidae, does not yet have a complete mitochondrial genome. Therefore, for the first time, this study provided the complete mitochondrial genomes of two species (*Meligethinus tschungseni* and *Brassicogethes affinis*) of Meligethinae, with a detailed annotation and analysis of their sequences. There is also another complete mitochondrial genome sequence of Meligethinae (*Teucriogethes* sp.) that has been uploaded to GenBank by authors but not yet published. These four subfamilies (Nitidulinae, Carpophilinae, Epuraeinae, Meligethinae) for which mitochondrial genome data are available so far are the most species-rich groups in Nitidulidae, accounting for approximately 3/4 of the total species in Nitidulidae [1,4,7]. Therefore, it is possible to further analyze the status of Nitidulidae and the sister group relationship of Nitidulidae. Moreover, it is necessary to preliminarily explore the status and the sister group relationship of Meligethinae based on the complete mitochondrial genomes for the first time. In this study, we reconstructed the phylogenetic relationships of 20 species (including 17 ingroups and 3 outgroups) under the site-heterogeneous mixture CAT + GTR substitution model (BI trees) and the best model (ML trees), respectively.

## 2. Materials and Methods

### 2.1. Sample Preparation and DNA Extraction

Adults of *M*. *tschungseni* in this study were collected from palm flowers and *B*. *affinis* from rape flowers in April and May 2022 at the West Campus of Yangtze University, Jingzhou, Hubei, China. All the specimens were immediately preserved in absolute ethanol. The total genomic DNA was extracted using the Ezup Column Animal Genomic DNA Purification Kit (Sangon Biotech, Shanghai, China).

### 2.2. Sequence Analysis

The mitochondrial genomes of *M*. *tschungseni* and *B*. *affinis* were sequenced using next-generation sequencing (NGS; Illumina NovaSeq6000; Berry Genomics, Beijing, China). The raw paired reads were trimmed and assembled using Geneious 8.1.3 (Biomatters, Auckland, New Zealand) with default parameters [50]. The complete mitochondrial genome of *Carpophilus pilosellus* Motschulsky, 1858 (Nitidulidae: Carpophilinae; NC_046035) [47] was selected as the reference sequence.

The positions of 13 protein-coding genes (PCGs) were determined by finding ORFs based on the invertebrate mitochondrial genetic codon and comparing with reference sequences. The positions of 22 tRNAs were determined according to the prediction results of the MITOS Web Server (http://mitos.bioinf.uni-leipzig.de/index.py (accessed on 13 May 2022)) [51]. The secondary structures of 22 tRNAs were predicted according to MITOS and the tRNAscan-SE Online Search Server [52] and then drawn using Adobe Illustrator CS5. The positions of rRNAs (*rrnL* and *rrnS*) and the control region (A + T-rich region) were determined based on the positions of the tRNAs and comparison with other homologous sequences. Tandem repeats in the control region were determined using the Tandem Repeats Finder Online server (http://tandem.bu.edu/trf/trf.html (accessed on 23 May 2022)) [53]. Circular maps of the mitochondrial genome were drawn using Organellar Genome DRAW (OGDRAW) (https://chlorobox.mpimp-golm.mpg.de/OGDraw.html (accessed on 16 May 2022)) [54]. The base composition, AT and GC skew, and relative synonymous codon usage (RSCU) of 10 species of Nitidulidae were calculated using PhyloSuite v1.2.2 [55]. The nucleotide diversity (Pi) of 13 PCGs, 22 tRNAs, and 2 rRNAs of 10 species of Nitidulidae were calculated using DnaSP v6.0 [56] with a step size of 20 bp and a sliding window of 200 bp. The non-synonymous (Ka)/synonymous (Ks) mutation rate ratios for 13 PCGs of 10 species of Nitidulidae were also calculated using DnaSP v6.0 [56]. The genetic distances between the mitochondrial genomes of 10 species of Nitidulidae were calculated using MEGA-X [57] based on the Kimura-2-parameter (K2P) model.

### 2.3. Phylogenetic Analysis

In this study, we analyzed the phylogenetic relationships among 20 species of Coleoptera based on the complete mitochondrial genomes. The information regarding these 20 species is shown in Table 1. Two newly sequenced mitochondrial genomes (*M. tschungseni* and *B. affinis*) were provided and analyzed in this study, one mitochondrial genome (*Teucriogethes* sp. from China) was sequenced and uploaded to GenBank by authors but has not yet been published, and the remaining mitochondrial genomes were downloaded from GenBank. Firstly, 13 PCGs and two rRNAs of these 20 species were aligned using Mafft v7.313 (PCG alignment strategy: G-INS-i; RNA alignment strategy: Q-INS-i). Secondly, poorly aligned and highly scattered regions were removed using Gblocks v0.91b. Then, the aligned and modified sequences were concatenated using PhyloSuite. Phylogenetic trees were constructed based on four datasets: (1) the first and second codon positions of 13 PCGs (PCG12); (2) all three codon positions of 13 PCGs (PCG123); (3) the first and second codon positions of 13 PCGs and two rRNAs (PCG12R); (4) all three codon positions of 13 PCGs and two rRNAs (PCG123R).

BI trees were established under the site-heterogeneous mixture CAT + GTR substitution model using PhyloBayes MPI v1.5a, running four Markov Chain Monte Carlo (MCMC) independently. When the sampled tree had stabilized and the four runs had reached satisfactory convergence (maxdiff < 0.3), the first 25% of the samples were discarded as “burn-in”. The ML trees were constructed using IQ-TREE v1.6.8 [61]. ModelFinder was used to select the substitution models (Table S2) for the ML analysis. A “greedy” algorithm and BIC (Bayesian information criterion) [55] were used to obtain the best model and optimal partitioning strategy for each partition. The ML analysis was performed using ultrafast bootstrap parameters of 1000 repetitions. The phylogenetic trees were visualized and edited using iTOL [62].

## 3. Results and Discussion

### 3.1. Genome Structure and Base Composition

**Figure 1:**
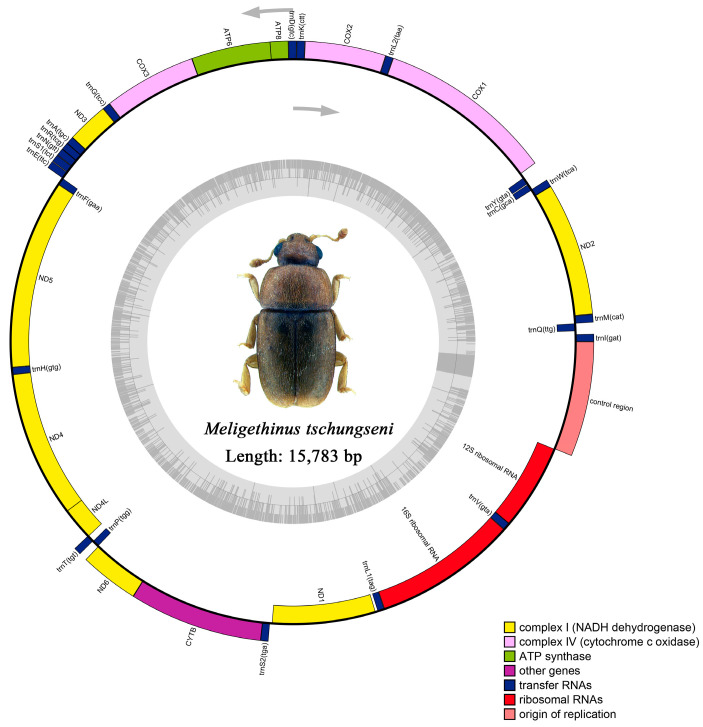
Circle map of the complete mitochondrial genome of *Meligethinus tschungseni*.

**Figure 2:**
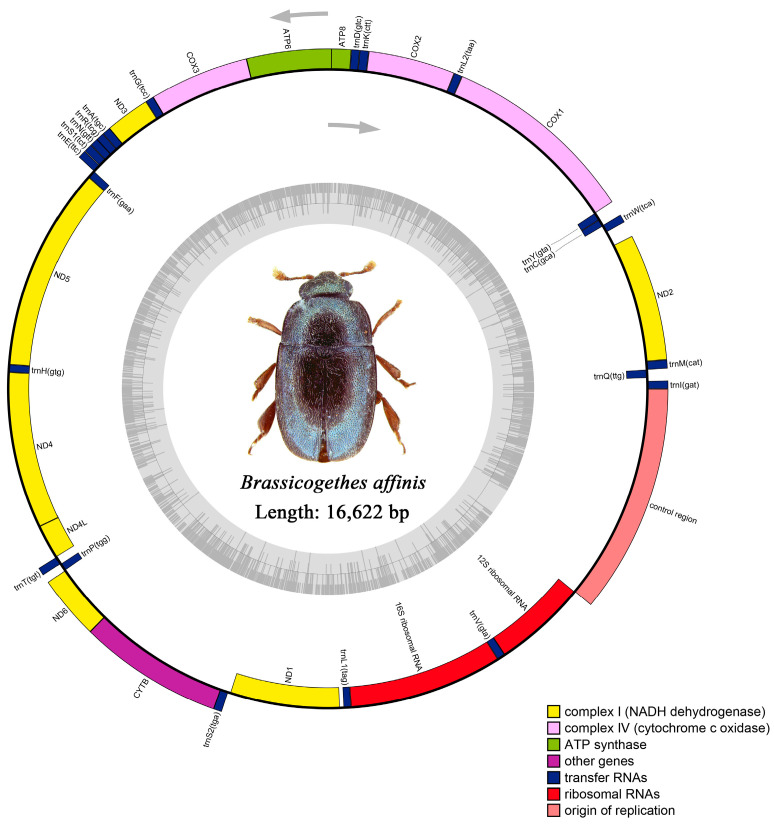
Circle map of the complete mitochondrial genome of *Brassicogethes affinis*.

The raw data of *M*. *tschungseni* and *B*. *affinis* were 6.21 gb and 4.65 gb, respectively. The complete mitochondrial genomes of *M. tschungseni* (GenBank accession number: ON782471) and *B. affinis* (GenBank accession number: ON782472) were 15,783 bp (Figure 1) and 16,622 bp (Figure 2), respectively. These two mitochondrial genomes showed the same gene arrangement as the other mitochondrial genomes of Nitidulidae. They contained the complete set of 37 genes (13 PCGs, 22 tRNAs, and 2 rRNAs) and a control region (A + T-rich region). The differences in the sequence length among Nitidulidae are mainly determined by the length of the control region and the length of the intergenic spacers between some tRNAs. The majority strand (J-strand) encoded most of the genes, including 9 PCGs (*nad2*, *cox1*, *cox2*, *atp8*, *atp6*, *cox3*, *nad3*, *nad6*, and *cytb*) and 14 tRNAs (*trnI*, *trnM*, *trnW*, *trnL2*, *trnK*, *trnD*, *trnG*, *trnA*, *trnR*, *trnN*, *trnS1*, *trnE*, *trnT*, and *trnS2*), while the minority strand (N-strand) encoded other genes, including 4 PCGs (*nad5*, *nad4*, *nad4L*, and *nad1*), 8 tRNAs (*trnQ*, *trnC*, *trnY*, *trnF*, *trnH*, *trnP*, *trnL1*, and *trnV*), and 2 rRNAs (*rrnL* and *rrnS*) (Table 2). Additionally, seven intergenic spacers were found in the mitochondrial genomes of *M. tschungseni* (113 bp in total) and *B. affinis* (200 bp in total) (Table 2). The longest intergenic spacer in *M. tschungseni* was between *trnY* and *cox1* (41 bp), and the longest in *B. affinis* was between *nad2* and *trnW* (122 bp) (Table 2). A total of 13 and 12 overlapping regions were found in the mitochondrial genomes of *M. tschungseni* (36 bp in total) and *B. affinis* (26 bp in total), respectively, and the longest overlapping regions were between *trnW* and *trnC* (8 bp), between *nad4L* and *trnT* (8 bp) in *M. tschungseni*, and between *trnW* and *trnC* (8 bp) in *B. affinis* (Table 2).

The AT contents of the mitochondrial genomes of *M. tschungseni* and *B. affinis* were 77% and 76.7%, respectively (Table 3 and Table 4), which were significantly higher than the GC content (Table 4). In addition, most of the known species of Nitidulidae showed a positive AT skew and negative GC skew in the mitochondrial genomes (Table 3), and *M*. *tschungseni* and *B*. *affinis* in this study also showed a positive AT skew and negative GC skew (Table 3 and Table 4), which indicated a higher content of A than T and a higher content of C than G in the mitochondrial genomes (Table 4).

### 3.2. Protein-Coding Genes (PCGs) and Codon Usage

The total lengths of 13 PCGs in the mitochondrial genomes of *M. tschungseni* and *B. affinis* were 11,196 bp and 11,097 bp, respectively (Table 4), both of which contained seven NADH dehydrogenase subunits (*nad1*, *nad2*, *nad3*, *nad4*, *nad5*, *nad6*, *nad4L*), three cytochrome c oxidase subunits (*cox1*, *cox2*, *cox3*), two ATPase subunits (*atp6*, *atp8*), and one cytochrome b gene (*cytb*) (Figure 1 and Figure 2, Table 5). The AT contents of 13 PCGs of *M. tschungseni* and *B. affinis* were 77.2% and 75.8%, respectively (Table 4). And both species showed a negative AT skew and a negative GC skew (Table 4), which indicated a higher content of T than A and a higher content of C than G. The AT contents of the third codon (91.1%, 87.4%) of *M. tschungseni* and *B. affinis* were much higher than that of the first codon (71.9%, 70.9%) and the second codon (68.7%, 68.8%) (Table 4). Other than *nad1* in the mitochondrial genomes of *Epuraea guttata*, *Omosita colon*, and *Aethina tumida* starting with TTG, the other PCGs of Nitidulidae in this study were typical start codons ATN (ATA, ATT, ATG, and ATC) (Table 5). Except for *cox1*, *cox2*, and *nad5*, all 10 species of Nitidulidae terminated with a single T, *cox3* and *nad4* always terminated with a single T, and *atp8* in *Xenostrongylus variegatus* terminated with a single T. The other PCGs in the mitochondrial genomes of the 10 species of Nitidulidae in this study all terminated with a stop codon TAA or TAG. Among them, *cox1*, *cox2*, *nad4*, and *nad5* in the mitochondrial genomes of *M*. *tschungseni* and *B*. *affinis* also terminated with a single T (Table 5). This incomplete stop codon is common in insects and can be converted to a complete stop codon through post-transcriptional polyadenylation [63].

**Figure 3:**
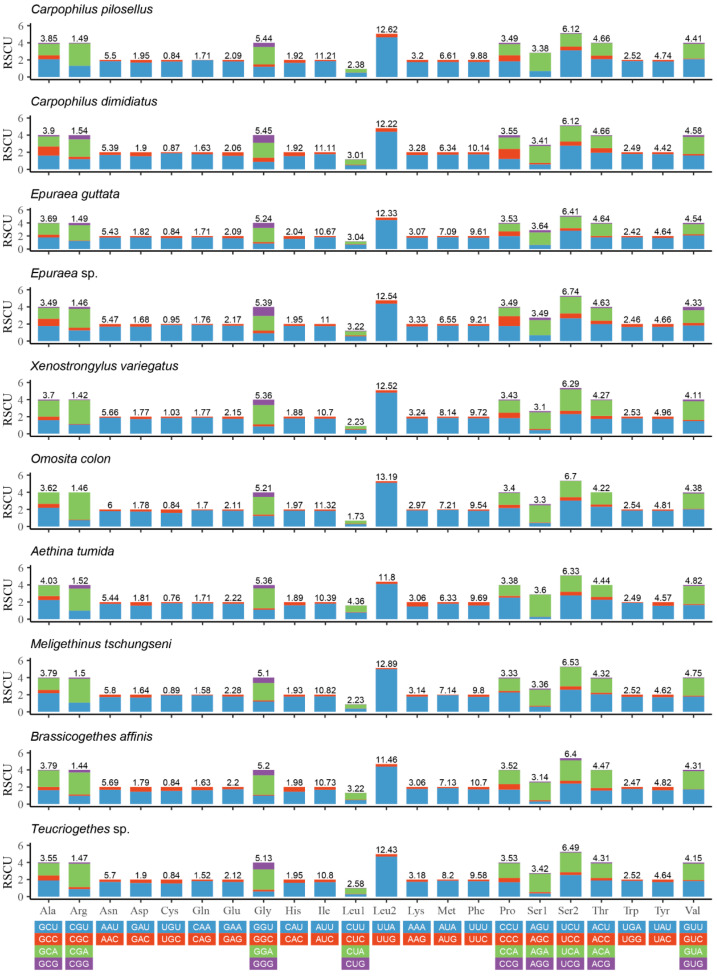
Relative synonymous codon usage (RSCU) of the PCGs of 10 species of Nitidulidae. The numbers above the bar graph indicate the frequency of amino acids.

The relative synonymous codon usage (RSCU) of PCGs of the known 10 species of Nitidulidae is shown in Figure 3. UUA (Leu2), AUU (Ile), UUU (Phe), and AUA (Met) were commonly used codons in Nitidulidae. These codons all consisted of A or U (Figure 3), which may be one of the reasons for the higher AT contents of the PCGs in Nitidulidae (Table 3).

### 3.3. Transfer and Ribosomal RNA Genes

**Figure 4:**
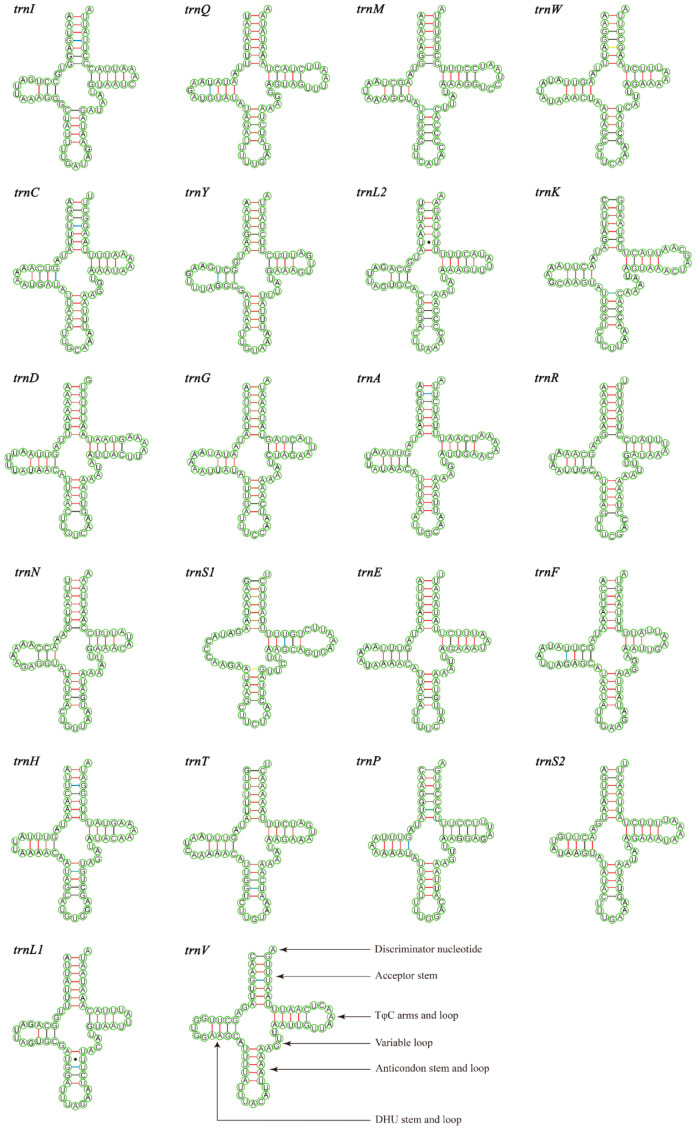
Predicted secondary structure for the tRNAs of *Meligethinus tschungseni* (A-U, G-C regular paired keys marked with red and black lines, respectively; G-U, C-U, A-C, A-G mismatched keys marked with blue, green, gray, and yellow lines, respectively; U-U mismatched keys marked with solid black dots).

**Figure 5:**
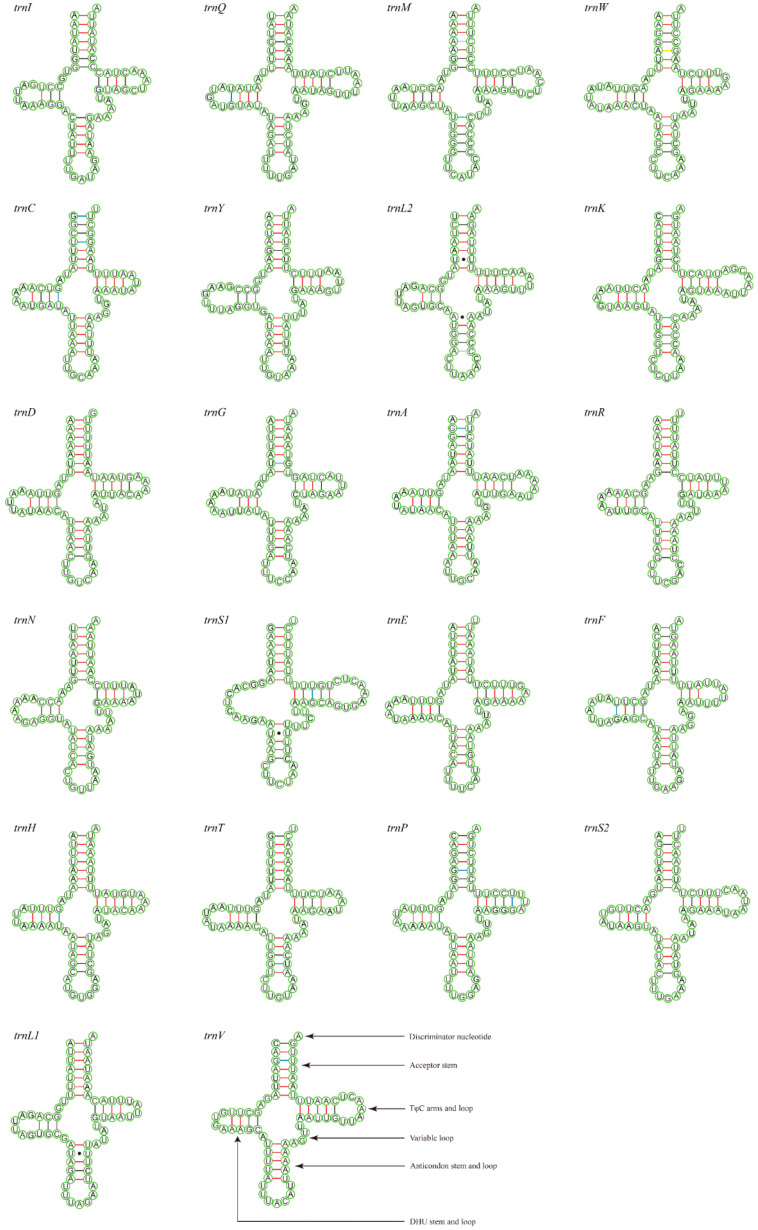
Predicted secondary structure for the tRNAs of *Brassicogethes affinis* (A-U, G-C regular paired keys marked with red and black lines, respectively; G-U, C-U, A-C, A-G mismatched keys marked with blue, green, gray, and yellow lines, respectively; U-U, A-A mismatched keys marked with solid black dots).

The total lengths of the 22 tRNAs of *M*. *tschungseni* and *B*. *affinis* were 1455 bp and 1451 bp, respectively; within the known tRNA length range of the mitochondrial genomes of Nitidulidae (Table 3 and Table 4). The AT contents of the tRNAs of *M*. *tschungseni* and *B*. *affinis* were 78% and 79%, respectively (Table 3 and Table 4). In addition, the tRNAs of both species showed a positive AT skew and positive GC skew (Table 4), indicating more A than T and more G than C. Except for *trnS1*, which showed a reduced dihydrouridine (DHU) arm, the other 21 tRNAs of *M*. *tschungseni* and *B*. *affinis* consisted of “four arms” and “four loops”, they could fold into the typical clover-leaf structure, and the amino-acid arm (14 bp) and the anticodon loop (7 bp) were highly conserved (Figure 4 and Figure 5). The DHU arm had three or four base pairs and the TφC arm had 3–5 base pairs in both *M*. *tschungseni* and *B*. *affinis* (Figure 4 and Figure 5). The lengths of the DHU loop in *M. tschungseni* and *B. affinis* were 3–8 bases and 4–8 bases, respectively. The lengths of the TφC loop of both *M*. *tschungseni* and *B*. *affinis* were 3–9 bases (Figure 4 and Figure 5). There were five types (G-U, C-U, A-C, A-G, U-U) of a total of 24 mismatched base pairs in *M. tschungseni* and six types (G-U, C-U, A-C, A-G, U-U, A-A) of 24 mismatched base pairs in *B. affinis* (Figure 4 and Figure 5).

In *M. tschungseni* and *B. affinis*, the total lengths of the two rRNAs were 2073 bp and 2075 bp, respectively (Table 3 and Table 4). The *rrnL* were all 1292 bp in these two species and were located between *trnL1* and *trnV*. The *rrnS* were 781 bp and 783 bp, respectively, and were located between *trnV* and the control region (Figure 1 and Figure 2, Table 2). The AT contents of the rRNAs of *M*. *tschungseni* and *B*. *affinis* were both high, 81.5% and 79.3%, respectively, and both showed a negative AT skew and positive GC skew, indicating a higher content of T than A and a higher content of G than C (Table 3 and Table 4).

### 3.4. Control Region

**Figure 6:**
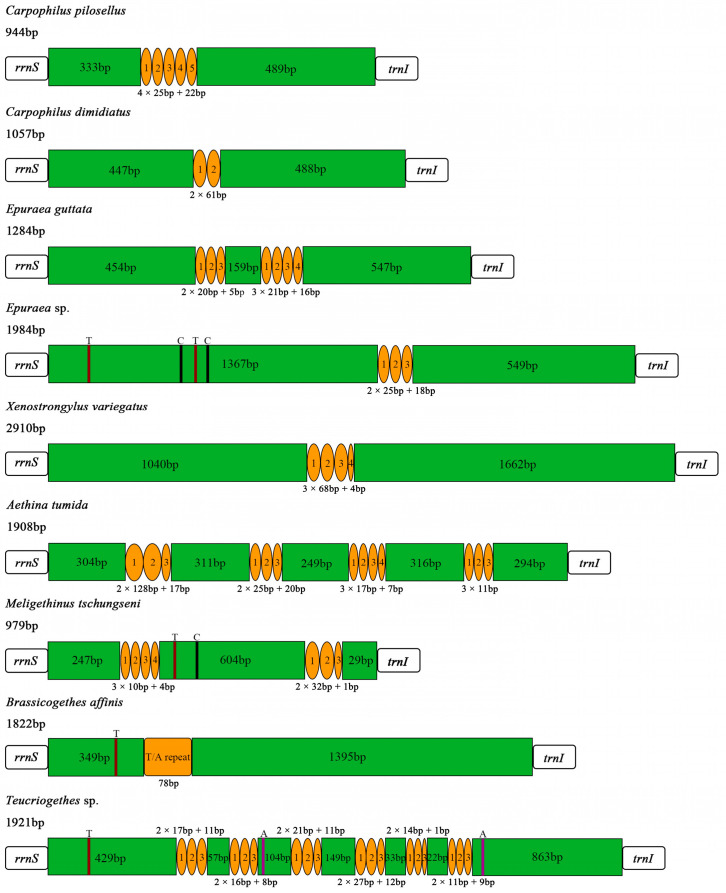
Structure of the control regions in the Nitidulidae mitochondrial genomes. Orange circles and box represent tandem repeat regions, and green boxes represent non-repeat regions. The brown, black, and purple regions represent poly (T), poly (C), and poly (A), respectively.

The control region (A + T-rich region) was located between *rrnS* and *trnI,* with lengths of 979 bp and 1822 bp in *M. tschungseni* and *B. affinis*, respectively (Figure 1 and Figure 2, Table 2, Table 3 and Table 4), within the known control region lengths of the mitochondrial genomes of Nitidulidae (Table 3) [64]. The AT contents were 62.5% and 76.1%, respectively (Table 3 and Table 4). Both species showed a negative AT skew and negative GC skew (Table 4), indicating a higher content of T than A and a higher content of C than G. The number of tandem repeat regions in the control region greatly varied among the 10 species of Nitidulidae mitochondrial genomes. Among them, *Omosita colon* had no tandem repeat region, *Carpophilus pilosellus*, *C. dimidiatus*, *Epuraea* sp., *Xenostrongylus variegatus*, and *B. affinis* had one tandem repeat region, *E. guttata* and *M. tschungseni* had two tandem repeat regions, and *Aethina tumida* and *Teucriogethes* sp. had four and six tandem repeat regions, respectively (Figure 6).

### 3.5. Nucleotide Diversity and Genetic Distance

**Figure 7:**
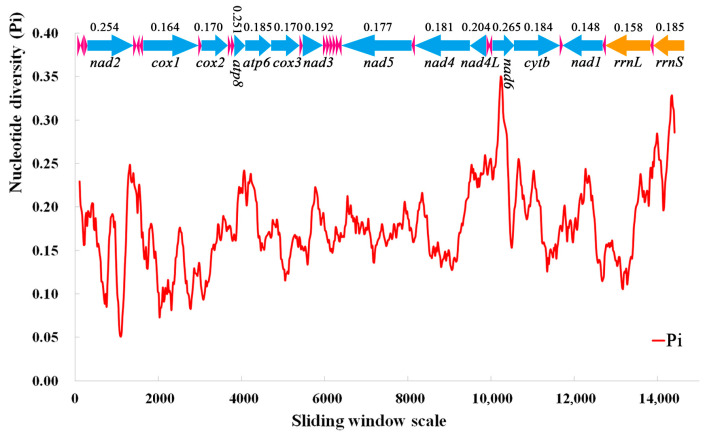
Sliding window analysis of 13 PCGs, 22 tRNAs, and 2 rRNAs in the mitochondrial genomes of 10 species of Nitidulidae. The red line represents the nucleotide diversity (Pi) value (window size = 200 bp, step size = 20 bp); the arrows represent the direction of gene coding—above the arrow is the Pi value of each gene, and below the arrow is the name of each gene; the blue arrows represent 13 PCGs, the pink arrows represent 22 tRNAs, and the orange arrows represent two rRNAs.

A sliding window analysis was used to study the nucleotide diversity of 13 PCGs, 22 tRNAs, and 2 rRNAs in the mitochondrial genomes of 10 species of Nitidulidae (Figure 7). The nucleotide diversity values ranged from 0.148 (*nad1*) to 0.265 (*nad6*). The *nad6* (0.265), *nad2* (0.254), and *atp8* (0.251) genes had a higher nucleotide diversity, indicating that these genes had a high variability in Nitidulidae. On the contrary, *nad1* (0.148) and *cox1* (0.164) had a lower nucleotide diversity; therefore, *nad1* and *cox1* were conserved genes in Nitidulidae.

**Figure 8:**
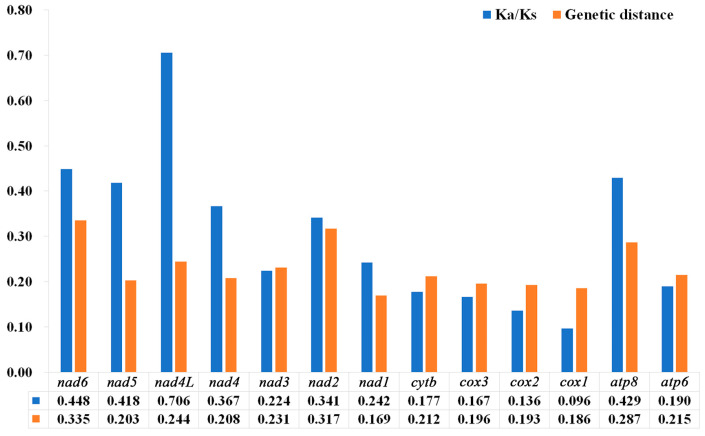
Genetic distances and ratios of non-synonymous (Ka) to synonymous (Ks) substitution rates of 13 PCGs among 10 species of Nitidulidae. The average value for each PCG is shown below the gene name.

The results showed that the Ka/Ks values of the 13 PCGs were between 0.096–0.706, and the Ka/Ks values were all less than 1 (Figure 8), representing all the genes that evolved under purifying selection. Furthermore, *cox1* (0.096) had the lowest Ka/Ks value, the lowest evolution rate, and exhibited the strongest purifying selection. In contrast, *nad4L* (0.706) and *nad6* (0.448) showed higher Ka/Ks values than the other PCGs, and they exhibited relaxed purifying selection. The results of the pairwise genetic distances of the 13 PCGs of the 10 species of Nitidulidae are shown in Figure 8. *cox1* (0.186) and *nad1* (0.169) evolved relatively slowly, while *nad6* (0.335) and *nad2* (0.317) evolved relatively quickly.

A nucleotide diversity analysis is the key to designing species-specific markers, and it aids in the molecular identification of species that are difficult to identify based on morphology [65,66,67]. Generally, *cox1* can be used as a potential marker for species identification and has been widely used in insect classification [68]. While in this study, *cox1* was the most conserved gene in the mitochondrial genomes of Nitidulidae, *nad6* had the fastest evolutionary rate compared with the other PCGs. Therefore, *nad6* may be more suitable as a barcode gene for species identification of Nitidulidae.

### 3.6. Phylogenetic Analysis

**Figure 9:**
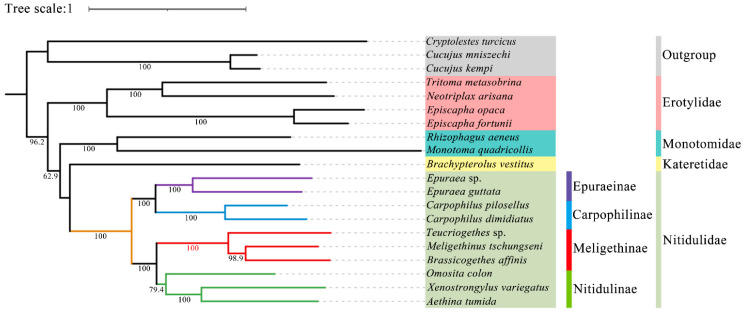
Phylogenetic tree generated based on the ML analysis of the PCG123 dataset under the best model. The bootstrap support values of the corresponding nodes are represented by Arabic numerals.

**Figure 10:**
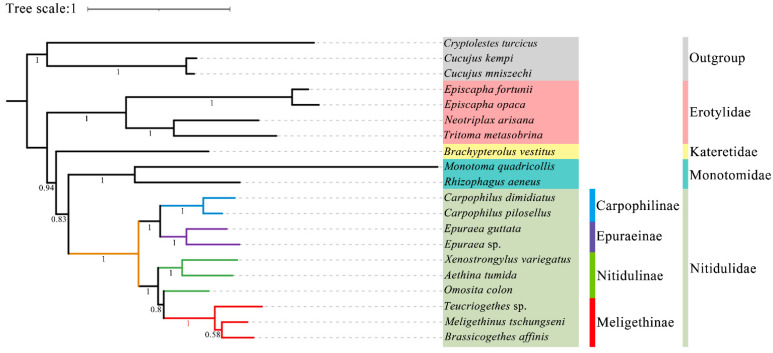
Phylogenetic tree generated based on the PhyloBayes analysis of the PCG123 dataset under the site-heterogeneous mixture CAT + GTR substitution model. The Bayesian posterior probabilities of the corresponding nodes are represented by Arabic numerals.

In this study, all the sites of the nucleotide substitution saturation test (Table S1) in the Gblocks showed that the index of substitution saturation (*Iss*) was less than the critical *Iss* based on a symmetrical tree (*Iss.cSym*) and *p* < 0.05. Table S2 lists the best model and optimal partitioning strategy selected for the four datasets of the ML analysis by ModelFinder. Four datasets (PCG12, PCG123, PCG12R, PCG123R) were used to construct ML trees and PhyloBayes trees for 20 species of *Coleoptera*, and a total of eight phylogenetic trees were obtained (Figure 9 and Figure 10 and Figures S1–S6).

Although the topological structure of these eight trees are not exactly same, they all strongly support (ML bootstrap support values (BS) = 100, Bayesian posterior probabilities (BPP) = 1) that the four subfamilies (Nitidulinae, Meligethinae, Carpophilinae, Epuraeinae) of Nitidulidae formed a monophyletic group based on the available complete mitochondrial genomes (Figure 9 and Figure 10 and Figures S1–S6). We further assumed that Nitidulidae is monophyletic, which is consistent with the results of studies based on morphological and molecular data analysis [1,3,27,28,29,30]. Regarding the sister group relationship of Nitidulidae, based on the PCG123 dataset analyzed using ML, the topological structure of the tree supported that the sister group relationship of Nitidulidae is Nitidulidae + Kateretidae (Figure 9), which is consistent with previous studies based on morphological characteristics [7,31,32], short molecular fragments [2,25,28], and fossil data [3]. Another seven topological trees showed that the sister group relationship of Nitidulidae is Nitidulidae + Monotomidae, but this clade has a BS of < 69 and BPP of < 0.85 (Figure 10 and Figures S1–S6). Previous studies have shown that clades with a of BS 50–69 or BPP of 0.85–0.89 are considered weakly supported, and clades with a BS of < 50 or BPP of < 0.85 are considered unsupported [69]; therefore, the sister group relationship of Nitidulidae + Monotomidae is untenable. Previously, Chen et al. [29] first proposed a sister group relationship of Nitidulidae + Monotomidae based on the complete mitochondrial genomes of 17 species. However, this clade had a low BS in ML trees. This study further supported that the sister group relationship of Nitidulidae is Nitidulidae + Kateretidae; however, considering that there is only one complete mitochondrial genome sequence of Kateretidae in GenBank, in future studies, we will strive to sequence more mitochondrial genomes of Kateretidae and Nitidulidae to explore the sister group relationship of Nitidulidae more clearly.

More importantly, although the mitochondrial genomes of all the genera in Meligethinae have not yet been obtained, this study attempted to analyze the taxonomic status and sister group relationship of Meligethinae based on the complete mitochondrial genomes of three genera and three species. The topological structures of all eight trees (Figure 9 and Figure 10 and Figures S1–S6) clearly showed that three genera (*Meligethinus*, *Brassicogethes*, *Teucriogethes*) of Meligethinae clustered into a single clade. Therefore, we further assumed that Meligethinae is monophyletic (BS = 100, BPP = 1), which is consistent with the studies based on adult morphological characteristics, biological habits, and short molecular fragments by Kirejtshuk et al. [34,35,36,37], Trizzino et al. [38], Audisio et al. [39], and Lee et al. [1]. Regarding the sister group relationship of Meligethinae, four ML trees supported (BS = 100) the sister group relationship of Meligethinae + Nitidulinae (Figure 9 and Figures S1–S3). However, the four BI trees showed that the zoosaprophagous *Omosita colon* [70] and the anthophagous Meligethinae [71] were abnormally clustered into one clade (Figure 10 and Figures S4–S5), but the BPP of this clade was <0.85, so this clade was considered unsupported [69]. In future studies, we plan to add new sequences of representative species of each subfamily of Nitidulidae, as well as of different and more distantly related genera and complexes of genera within Nitidulinae, to further explore the phylogenetic relationships of Meligethinae.

## 4. Conclusions

In this study, two complete mitochondrial genomes of Meligethinae (*Meligethinus tschungseni* and *Brassicogethes affinis*) were provided for the first time, and the mitochondrial genomes of 10 species among Nitidulidae were compared. The phylogenetic trees of 20 species of related families of Coleoptera were constructed to try to analyze the higher phylogeny of Nitidulidae. Based on the available complete mitochondrial genomes, this study confirmed that the four subfamilies (Nitidulinae, Meligethinae, Carpophilinae, Epuraeinae) of Nitidulidae formed a monophyletic group, further supporting that the sister group relationship of Nitidulidae is Nitidulidae + Kateretidae. This study also assumed that Meligethinae is monophyletic (BS = 100, BPP = 1) based on the complete mitochondrial genome, which was analyzed for the first time, and its sister group relationship is likely to be Meligethinae + Nitidulinae. 

Considering that the representative genera used in this study do not cover all genera, in the future, it is necessary to sequence the complete mitochondrial genomes of more species for an in-depth molecular phylogenetic analysis of Nitidulidae. Furthermore, the phylogenetic analysis of Nitidulidae can be based on an integrative approach, such as combining the morphological characteristics of adults and larvae, mitochondrial genome data, nuclear genome data, and fossils, as well as biological information such as host plants, etc.

## Figures and Tables

**Table 1:** Summary of mitochondrial genome information used in this study.

Family	Subfamily	Species	Accession Number	Reference
Laemophloeidae		*Cryptolestes turcicus*	KT070712.1	[58]
Cucujidae		*Cucujus kempi*	NC_051939.1	[59]
		*Cucujus mniszechi*	NC_051938.1	[59]
Erotylidae		*Tritoma metasobrina*	MZ014622.1	[60]
		*Episcapha opaca*	MZ014623.1	[60]
		*Neotriplax arisana*	MZ014624.1	[60]
		*Episcapha fortunii*	NC_067051.1	Unpublished
Monotomidae		*Monotoma quadricollis*	NC_036266.1	Unpublished
		*Rhizophagus aeneus*	KX087340.1	Unpublished
Kateretidae		*Brachypterolus vestitus*	KX087245.1	Unpublished
Nitidulidae	Carpophilinae	*Carpophilus pilosellus*	NC_046035.1	[47]
		*Carpophilus dimidiatus*	NC_046036.1	[47]
	Epuraeinae	*Epuraea guttata*	KX087289.1	Unpublished
		*Epuraea* sp.	MW044619.1	[29]
	Nitidulinae	*Xenostrongylus variegatus*	MW044620.1	[29]
		*Omosita colon*	NC_050852.1	[48]
		*Aethina tumida*	NC_036104.1	[49]
	Meligethinae	*Meligethinus tschungseni*	ON782471	This study
		*Brassicogethes affinis*	ON782472	This study
		*Teucriogethes* sp.	OR387485	Unpublished

**Table 2:** Mitogenomic organization of *Meligethinus tschungseni* and *Brassicogethes affinis*.

Gene	Position		Size (bp)	Intergenic Nucleotides	Codon		Strand
	From	To			Start	Stop	
*Meligethinus tschungseni*/*Brassicogethes affinis*							
*trnI*	1/1	65/65	65/65				+/+
*trnQ*	94/91	162/159	69/69	28/25			−/−
*trnM*	162/159	230/227	69/69	−1/−1			+/+
*nad2*	231/228	1364/1232	1134/1005		ATT/ATT	TAA/TAA	+/+
*trnW*	1363/1355	1428/1420	66/66	−2/122			+/+
*trnC*	1421/1413	1483/1474	63/62	−8/−8			−/−
*trnY*	1489/1457	1554/1540	66/66	5			−/−
*cox1*	1596/1538	3093/3083	1498/1546	41/−3	ATC/ATT	T/T	+/+
*trnL2*	3094/3084	3158/3148	65/65				+/+
*cox2*	3159/3149	3846/3836	688/688		ATC/ATC	T/T	+/+
*trnK*	3847/3837	3916/3907	70/71				+/+
*trnD*	3917/3908	3985/3973	69/66				+/+
*atp8*	3986/3974	4141/4129	156/156		ATC/ATT	TAG/TAG	+/+
*atp6*	4138/4126	4809/4797	672/672	−4/−4	ATA/ATA	TAA/TAA	+/+
*cox3*	4809/4797	5597/5585	789/789	−1/−1	ATG/ATG	TAA/TAA	+/+
*trnG*	5598/5585	5662/5649	65/65	/−1			+/+
*nad3*	5663/5650	6016/6003	354/354		ATT/ATT	TAG/TAG	+/+
*trnA*	6015/6002	6081/6068	67/67	−2/−2			+/+
*trnR*	6081/6069	6144/6132	64/64	−1			+/+
*trnN*	6144/6132	6208/6195	65/64	−1/−1			+/+
*trnS1*	6209/6196	6275/6262	6767				+/+
*trnE*	6277/6264	6342/6329	66/66	1/1			+/+
*trnF*	6342/6329	6407/6394	66/66	−1/−1			−/−
*nad5*	6408/6395	8121/8108	1714/1714		ATC/ATC	T/T	−/−
*trnH*	8122/8109	8185/8172	64/64				−/−
*nad4*	8186/8173	9509/9490	1324/1318		ATA/ATT	T/T	−/−
*nad4L*	9506/9490	9802/9777	297/288	−4/−1	ATA/ATG	TAA/TAA	−/−
*trnT*	9795/9780	9860/9845	66/66	−8/2			+/+
*trnP*	9861/9846	9924/9909	64/64				−/−
*nad6*	9926/9911	10,429/10,417	504/507	1/1	ATT/ATT	TAA/TAA	+/+
*cytb*	10,429/10,417	11,565/11,559	1137/1143	−1/−1	ATG/ATG	TAG/TAG	+/+
*trnS2*	11,564/11,558	11,630/11,625	67/68	−2/−2			+/+
*nad1*	11,649/11,644	12,581/12,564	933/921	18/18	ATT/ATT	TAG/TAG	−/−
*trnL1*	12,601/12,596	12,663/12,658	63/63	19/31			−/−
*rrnL*	12,664/12,659	13,955/13,950	1292/1292				−/−
*trnV*	13,956/13,951	14,024/14,018	69/68				−/−
*rrnS*	14,024/14,018	14,804/14,800	781/783				−/−
control region	14,805/14,801	15,783/16,622	979/1822				

**Table 3:** Nucleotide composition of mitochondrial genomes of 10 species of Nitidulidae: *Carpophilus pilosellus* (C1.); *Carpophilus dimidiatus* (C2.); *Epuraea guttata* (E1.); *Epuraea* sp. (E2.); *Xenostrongylus variegatus* (X.); *Omosita colon* (O.); *Aethina tumida* (A.); *Meligethinus tschungseni* (M.); *Brassicogethes affinis* (B.); *Teucriogethes* sp. (T.).

Species	Whole Genome		AT Skew	GC Skew	PCGs		tRNAs		rRNAs		Control Region	
	Size (bp)	AT (%)			Size (bp)	AT (%)	Size (bp)	AT (%)	Size (bp)	AT (%)	Size (bp)	AT (%)
*C1.*	15,686	77.2	0.027	−0.177	11,103	76.5	1442	76.3	2079	77.5	944	86.8
*C2.*	15,717	75.2	0.038	−0.202	11,094	74.5	1441	74.9	2061	75	1057	83.5
*E1.*	16,021	76.5	0.043	−0.19	11,073	75.7	1451	75.7	2081	76.4	1284	85.0
*E2.*	16,641	76.4	−0.015	−0.216	11,100	74.9	1445	75.7	2081	78.8	1984	82.6
*X.*	17,657	77.2	0.021	−0.141	11,040	77	1454	78.2	2079	81.3	2910	74.7
*O.*	16,544	79.3	0.029	−0.178	11,127	77.9	1453	79.4	2083	82.2	645	86.1
*A.*	16,576	76.9	0.034	−0.223	11,109	75.4	1460	77.2	2064	79.5	1908	82.4
*M.*	15,783	77	0.029	−0.236	11,196	77.2	1455	78	2073	81.5	979	62.5
*B.*	16,622	76.7	0.061	−0.175	11,097	75.8	1451	79	2075	79.3	1822	76.1
*T.*	16,737	79.9	0.102	−0.165	11,082	76.5	1461	79.1	2099	81.2	1921	97.7

**Table 4:** Nucleotide composition of mitochondrial genomes of *Meligethinus tschungseni* and *Brassicogethes affinis*.

Regions	Size (bp)	T(U)	C	A	G	AT(%)	GC(%)	AT Skew	GC Skew
*Meligethinus tschungseni*									
Full genome	15,783	37.4	14.2	39.6	8.8	77	23	0.029	−0.236
PCGs	11,196	43.7	11.4	33.5	11.3	77.2	22.7	−0.132	−0.006
tRNAs	1455	36.8	9.8	41.2	12.2	78	22	0.056	0.106
rRNAs	2073	42.5	6.2	39	12.3	81.5	18.5	−0.042	0.328
1st codon position	3732	37.4	10.6	34.5	17.5	71.9	28.1	−0.04	0.248
2nd codon position	3732	47.5	17.9	21.2	13.3	68.7	31.2	−0.383	−0.147
3rd codon position	3732	46.3	5.8	44.8	3	91.1	8.8	−0.016	−0.317
Control region	979	41.2	34.4	21.3	3.1	62.5	37.5	−0.317	−0.837
*Brassicogethes affinis*									
Full genome	16,622	36	13.7	40.7	9.6	76.7	23.3	0.061	−0.175
PCGs	11,097	42.4	12.8	33.4	11.5	75.8	24.3	−0.119	−0.052
tRNAs	1451	37.6	9.2	41.4	11.9	79	21.1	0.047	0.128
rRNAs	2075	42	7.3	37.3	13.3	79.3	20.6	−0.058	0.291
1st codon position	3699	36.8	11.8	34.1	17.2	70.9	29	−0.038	0.189
2nd codon position	3699	47.4	18.1	21.4	13.1	68.8	31.2	−0.379	−0.163
3rd codon position	3699	42.8	8.4	44.6	4.2	87.4	12.6	0.02	−0.332
Control region	1822	44.1	12.2	32.0	11.7	76.1	23.9	−0.159	−0.021

**Table 5:** Start and stop codons of the mitochondrial genomes of 10 species of Nitidulidae: *Carpophilus pilosellus* (C1.); *Carpophilus dimidiatus* (C2.); *Epuraea guttata* (E1.); *Epuraea* sp. (E2.); *Xenostrongylus variegatus* (X.); *Omosita colon* (O.); *Aethina tumida* (A.); *Meligethinus tschungseni* (M.); *Brassicogethes affinis* (B.); *Teucriogethes* sp. (T.).

Gene	Start Codon/Stop Codon									
	*C1.*	*C2.*	*E1.*	*E2.*	*X.*	*O.*	*A.*	*M.*	*B.*	*T.*
*atp6*	ATA/TAA	ATG/TAA	ATG/TAA	ATG/TAA	ATA/TAA	ATG/TAA	ATA/TAA	ATA/TAA	ATA/TAA	ATA/TAA
*atp8*	ATC/TAG	ATC/TAG	ATT/TAG	ATC/TAG	ATC/T	ATT/TAG	ATT/TAG	ATC/TAG	ATT/TAG	ATC/TAG
*cox1*	ATT/T	ATT/T	ATT/T	ATC/T	ATT/T	ATT/T	ATT/T	ATC/T	ATT/T	ATA/T
*cox2*	ATT/T	ATC/T	ATA/T	ATT/T	ATT/T	ATT/T	ATT/T	ATC/T	ATC/T	ATT/T
*cox3*	ATG/T	ATG/T	ATG/T	ATG/T	ATG/T	ATG/T	ATG/T	ATG/TAA	ATG/TAA	ATG/TAA
*cytb*	ATG/TAG	ATG/TAG	ATA/TAG	ATG/TAG	ATG/TAG	ATG/TAG	ATG/TAA	ATG/TAG	ATG/TAG	ATG/TAA
*nad1*	ATG/TAG	ATA/TAG	TTG/TAG	ATT/TAG	ATT/TAG	TTG/TAG	TTG/TAG	ATT/TAG	ATT/TAG	ATA/TAG
*nad2*	ATT/TAA	ATT/TAA	ATT/TAA	ATT/TAA	ATT/TAA	ATT/TAA	ATT/TAA	ATT/TAA	ATT/TAA	ATT/TAG
*nad3*	ATT/TAG	ATT/TAG	ATA/TAG	ATT/TAG	ATT/TAG	ATT/TAA	ATA/TAG	ATT/TAG	ATT/TAG	ATT/TAG
*nad4*	ATG/T	ATG/T	ATG/TAA	ATA/T	ATT/T	ATG/T	ATG/T	ATA/T	ATT/T	ATA/T
*nad4L*	ATG/TAA	ATG/TAA	ATG/TAA	ATG/TAA	ATG/TAA	ATG/TAA	ATG/TAA	ATA/TAA	ATG/TAA	ATG/TAA
*nad5*	ATT/T	ATT/T	ATA/T	ATT/T	ATT/T	ATT/T	ATA/T	ATC/T	ATC/T	ATC/T
*nad6*	ATA/TAA	ATA/TAA	ATC/TAA	ATA/TAA	ATA/TAA	ATT/TAA	ATA/TAA	ATT/TAA	ATT/TAA	ATT/TAA

## Data Availability

The newly sequenced mitochondrial genome in this study has been uploaded to GenBank (ON782471, ON782472, OR387485).

## Supplementary Materials

The following supporting information can be downloaded at: https://www.mdpi.com/article/10.3390/insects15010057/s1, Table S1: Substitution saturation tests for PCGs, rRNAs, and tRNAs of mitochondrial genomes of Coleoptera; Table S2: The best model and optimal partitioning strategy selected for the four datasets of the ML analysis using ModelFinder; Figure S1: Phylogenetic tree produced from the ML analysis based on the PCG12 dataset; Figure S2: Phylogenetic tree produced from the ML analysis based on the PCG12R dataset; Figure S3: Phylogenetic tree produced from the ML analysis based on the PCG123R dataset; Figure S4: Phylogenetic tree produced from the PhyloBayes analysis based on the PCG12 dataset; Figure S5: Phylogenetic tree produced from the PhyloBayes analysis based on the PCG12R dataset; Figure S6: Phylogenetic tree produced from the PhyloBayes analysis based on the PCG123R dataset.
