# Supplementary material for: The First Two Complete Mitochondrial Genomes for the Subfamily Meligethinae (Coleoptera: Nitidulidae) and Implications for the Higher Phylogeny of Nitidulidae
Source: Insects. 2024 Jan 12;15(1):57. doi: 10.3390/insects15010057 (PMC10816600; doi:10.3390/insects15010057)
Supplement: Supplementary file 1 [file insects-15-00057-s001.zip › Table S2. The best model and optimal partitioning strategy selected for the four datasets of ML analysis by ModelFinder.pdf]

Table S2. The best model and optimal partitioning strategy selected for the four datasets of ML analysis by ModelFinder.

Dataset	Optimal Partition	Subset Partitions	Best Model
PCG12	Partition1	*atp6*, *cox1*, *cox2*, *cox3*	GTR+F+R4
	Partition2	*atp8*, *nad2*, *nad3*	GTR+F+I+G4
	Partition3	*cytb*	GTR+F+I+G4
	Partition4	*nad1*, *nad4L*, *nad4*, *nad5*	GTR+F+I+G4
	Partition5	*nad6*	TIM+F+G4
PCG123	Partition1	*atp6*_codon1, *cox1*_codon1, *cox2*_codon1, *cox3*_codon1, *cytb*_codon1	GTR+F+R3
	Partition2	*atp6*_codon2, *cox1*_codon2, *cox2*_codon2, *cox3*_codon2, *cytb*_codon2	TVM+F+I+G4
	Partition3	*atp6*_codon3	HKY+F+G4
	Partition4	*atp8*_codon1, *nad2*_codon1, *nad3*_codon1	TIM2+F+I+G4
	Partition5	*atp8*_codon2, *nad2*_codon2, *nad3*_codon2, *nad6*_codon2	TVM+F+G4
	Partition6	*atp8*_codon3	TPM2u+F+R2
	Partition7	*cox1*_codon3, *cox2*_codon3	HKY+F+R3
	Partition8	*cox3*_codon3	TIM2+F+G4
	Partition9	*cytb*_codon3	TN+F+G4
	Partition10	*nad1*_codon1, *nad4L*_codon1, *nad4*_codon1, *nad5*_codon1	TVM+F+I+G4
	Partition11	*nad1*_codon2, *nad4L*_codon2, *nad4*_codon2, *nad5*_codon2	GTR+F+I+G4
	Partition12	*nad1*_codon3, *nad4*_codon3	TVM+F+R3
	Partition13	*nad2*_codon3	TN+F+G4
	Partition14	*nad3*_codon3	TPM2+F+R2
	Partition15	*nad4L*_codon3	HKY+F+R2
	Partition16	*nad5*_codon3	TPM3+F+R3
	Partition17	*nad6*_codon1	TIM2+F+I+G4
	Partition18	*nad6*_codon3	HKY+F+G4
PCG12R	Partition1	*atp6*, *cox1*, *cox2*, *cox3*	GTR+F+R4
	Partition2	*atp8*, *cytb*, *nad3*	GTR+F+I+G4
	Partition3	*nad1*	GTR+F+I+G4
	Partition4	*nad2*	GTR+F+I+G4
	Partition5	*nad4L*, *nad4*, *nad5*	GTR+F+I+G4
	Partition6	*nad6*	GTR+F+I+G4
	Partition7	*rrnL*, *rrnS*	GTR+F+I+G4
PCG123R	Partition1	*atp6*_codon1, *cox1*_codon1, *cox2*_codon1, *cox3*_codon1, *cytb*_codon1	GTR+F+R3
	Partition2	*atp6*_codon2, *cox1*_codon2, *cox2*_codon2, *cox3*_codon2, *cytb*_codon2	TVM+F+I+G4
	Partition3	*atp6*_codon3	HKY+F+G4

Partition4	*atp8*_codon1, *nad2*_codon1, *nad3*_codon1	TIM2+F+I+G4
Partition5	*atp8*_codon2, *nad2*_codon2, *nad3*_codon2, *nad6*_codon2	TVM+F+G4
Partition6	*atp8*_codon3	TPM2u+F+R2
Partition7	*cox1*_codon3	HKY+F+R3
Partition8	*cox2*_codon3	HKY+F+R2
Partition9	*cox3*_codon3	TN+F+G4
Partition10	*cytb*_codon3	TN+F+G4
Partition11	*nad1*_codon1, *nad4L*_codon1, *nad4*_codon1, *nad5*_codon1, *rrnS*	TVM+F+I+G4
Partition12	*nad1*_codon2, *nad4L*_codon2, *nad4*_codon2, *nad5*_codon2	GTR+F+I+G4
Partition13	*nad1*_codon3, *nad4*_codon3	TPM3u+F+R3
Partition14	*nad2*_codon3	TN+F+G4
Partition15	*nad3*_codon3	TPM2u+F+R2
Partition16	*nad4L*_codon3	HKY+F+R2
Partition17	*nad5*_codon3	TPM3+F+R3
Partition18	*nad6*_codon1	TIM2+F+I+G4
Partition19	*nad6*_codon3	HKY+F+G4
Partition20	*rrnL*	GTR+F+I+G4
